# How mutant p53 empowers Foxh1 fostering leukaemogenesis?

**DOI:** 10.1038/s41420-019-0191-5

**Published:** 2019-06-24

**Authors:** Ivano Amelio

**Affiliations:** Medical Research Council, Toxicology Unit, Department of Pathology, University of Cambridge, Tennis Court Road, Cambridge, CB2 1QP UK

**Keywords:** Oncogenes, Cell growth

**Fig. 1:**
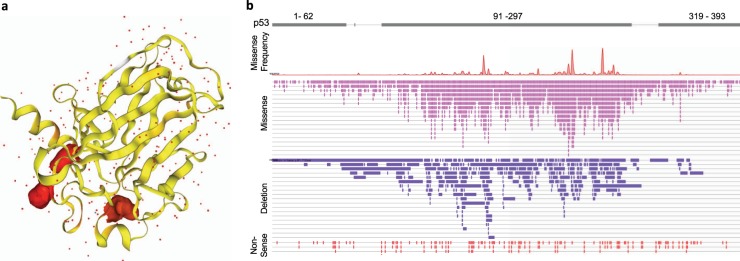
p53 mutational spectrum. **a** Structural representation of human p53 core domain with hot spot mutation R273H. **b** Schematic representation of p53 domains including information on frequencies and types of mutations spanning across the entire human p53 sequence in acute amyloid leukaemia. Red picks indicate frequency of hotspot mutations; pink bars indicate position and type of missense mutations; purple bars indicate position and type of in frame deletions; red bars indicate position and type of nonsense mutations. Highest frequency and variety of mutations is observed in the p53 DNA binding domain. Source: COSMIC mutation database

This year the biomedical research community is celebrating the 40th anniversary of the discovery of *the most frequently mutated gene across all human cancer*^1^. p53 was firstly identified in 1979; for a decade, its function was associated with its oncogenic properties, leading to the conclusion that p53 was a powerful oncogene^2–4^. Only later, the confusion was clarified: most of the researchers were unaware that they were in fact studying its mutant forms^5,6^. 40 years later, whether p53 mutants are effectively functioning as oncogenes and whether their gain-of-function effects are contributing to the pathogenesis of cancer remains largely controversial^7–9^ (Fig. 1). But how does mutant p53 gain its oncogenic properties? Scott Lowe’s group recently reported important observations in support of a major contribution of p53 mutants to myeloid leukaemia pathogenesis^10^.

In acute myeloid leukaemia (AML) *TP53* mutations are mainly associated with the subtype known as complex karyotype AML (CK-AML). CK-AML is a lethal disease (less than 2%, 5-year survival) characterised by the presence of several cytogenetic abnormalities. Lowe and colleagues’ work formally demonstrates that expression of p53^R172H^ (the mouse orthologue of the human R175H mutation) accelerates the onset of haematological malignancies beyond the effects of p53 deficiency^10^. Consistent with what has previously been reported, mice harbouring p53^R172H^ succumb from thymic lymphomas faster than mice completely lacking p53 expression. To assess the impact of p53^R172H^ in the clinically relevant context of AML, the authors transplanted bone marrow cells from *Mx1*-Cre;p53^R172H/F^ and *Mx1*-Cre;p53^F/F^ mice into thymectomized recipient mice. The p53^R172H/-^ mice succumbed to the disease faster than their p53-null counterparts. Importantly, depletion of p53^R172^ by an inducible shRNA system in AML cells led to differentiation and apoptosis, indicating that AML cells expressing p53^R172H^ acquire a molecular dependency on mutant p53. Hence, p53^R172^ contributes to a differentiation block that sustains leukemogenesis^10^. This set of data therefore supports a gain-of-function effect of p53^R172^ in an AML setting.

Lowe’s team observed that the adult bone marrow cells from p53^R172H/Δ^ mice have significantly higher replating capacity compared to cells from p53^-null^ mice in a serial replating experiment performed with limiting dilution cultures in methylcellulose medium. Consistent with these findings, competitive transplantation studies using p53^WT/WT^, p53^Δ/Δ^, and p53^R172H/Δ^ bone marrow cells demonstrated that p53^R172H/Δ^ cells outcompeted wild-type cells to a greater extent than p53^Δ/Δ^ cells. These observations establish a role for p53^R172H^ in a pre-malignant setting in sustaining pathologic self-renewal in adult hematopoietic cells. Remarkably, the increase in self-renewal capacity was observed in p53^R172H/Δ^ but not p53^R172H/WT^ cells, suggesting that loss of the residual WT allele is a prerequisite^10^. While overall these data support a p53^R172H^ gain-of-function effect as they contradict the long-lasting hypothesis of a dominant negative effect of p53 mutants on wt p53 (and possibly other p53 family members). This highlights the complexity of the p53 mutant gain-of-function effects and the corresponding difficulties in unifying diverse observations.

From a mechanistic standpoint, the authors showed that p53^R172H^ promotes expression of the Foxh1 transcriptional factor, thus supporting a transcriptional reprogramming that sustains the enhanced self-renewal phenotype of leukaemic cells. This transcriptional signature was also found to be correlated with p53 mutant status in human CK-AML. Foxh1 expression was proved to be necessary and sufficient to sustain the p53^R172H^-dependent phenotype. Enforced or reduced expression of Foxh1 affected hematopoietic cell differentiation and self-renewal capacity consistent with the pathological role of the p53^R172H^-Foxh1 axis^10^. Hence, this study demonstrates that, in this specific context, mutant p53 acts as a bona fide oncogene that contributes to the pathogenesis of CK-AML with a mechanism involving Foxh1.

In the wide range of proposed p53 mutant gain-of-function mechanisms and the highly diverse sets of observations related to its oncogenic phenotype, this study establishes proliferative potential and self-renewal capacity as consistent and relevant aspects in the biology of mutant p53 gain-of-function. Cancer cells expressing p53 mutants appear to acquire proliferative benefits that may substantially exceed the advantage conferred by loss of the wild-type endogenous p53. However, this simplistic interpretation of the mutant p53 gain-of-function does not necessarily explain how cancer cells became dependent on mutant p53 expression, a phenomenon highly reproducible across many cancer types and characteristic of several different mutants^11^. A proliferative advantage indeed does not fully justify the addiction that cancer cells display to p53 mutant expression. Despite its important therapeutic implications, the underlining mechanisms associated with p53 mutant dependency therefore remain largely unexplained.

It is also still unclear how mutant p53 is executing its gain-of-function effects. Is the p53 mutant protein capable of specifically controlling molecular signalling or is it randomly altering physiological molecular networks? p53^R172H^ leukaemic cells express high levels of Foxh1, but how this occurs remains to be determined. Several transcriptional factors, including SREBPs and HIF-1 have been shown to be altered in their transcriptional ability by mutant p53^12–14^, although it remains unclear how mutant p53 modifies their function. A fascinating unifying hypothesis could be that p53 status influences the global epigenetic landscape thus indirectly influencing the function of many transcriptional factors.

Additional work, and also careful reanalysis of the available data, is still required to assess the individual contribution of gain-of-function and loss-of-function mutant p53 to cancer pathogenesis. For example, it is interesting that loss of genomic integrity related to p53 inactivation is observed both in p53-null and p53 mutant backgrounds^12^. Genomic instability is a hallmark of malignant cancers and is crucially associated with the acquisition of the cellular plasticity that is necessary for evolution of the malignant disease. In assessing the relative contribution of the gain-of-function vs. the loss-of-function mutants, the contribution of any additional p53 mutant property might therefore appear marginal in comparison with the loss of genomic integrity. However, the strong selective pressure that leads to acquisition of missense mutations in the *TP53* gene rather than total gene deletion remains a very important argument in support of the relevance of the gain-of-function mutants^15^.
